# The Role of Regulatory B Cell-Like Malignant Cells and T_reg_ Cells in the Mouse Model of BCL1 Tumor Dormancy

**DOI:** 10.1371/journal.pone.0167618

**Published:** 2016-12-13

**Authors:** Andrew BitMansour, Laurentiu M. Pop, Ellen S. Vitetta

**Affiliations:** Department of Immunology and Cancer Immunobiology Center University of Texas Southwestern Medical Center at Dallas, Texas, United States of America; McGill University Health Centre, CANADA

## Abstract

Cancer dormancy is a clinical state in which residual tumor cells persist for long periods of time but do not cause detectable disease. In the mouse B cell lymphoma model (BCL1), dormancy can be induced and maintained by immunizing mice with a soluble form of the IgM expressed on the surface of the tumor cells. Immunization induces an anti-idiotype antibody response that maintains dormancy. Mice with dormant tumor have low numbers of BCL1 cells in their spleens that divide and are killed at the same rate. When the anti-Id antibodies wane, the tumor cells grow rapidly and kill the host. Spleens from tumor-bearing mice contain both effector (CD4^+^ and CD8^+^) and regulatory T cells (T_regs_). In other tumor models, it has been reported that T_regs_ promote tumor progression by preventing effector cells from killing the tumor. In this report, we demonstrate that the tumor site with rapidly dividing BCL1 cells has fewer T_regs_ than the tumor site harboring dormant BCL1 cells. In both cases, the T_regs_ were equally suppressive *in vitro*. In spleens from mice with actively growing tumor, CD8^+^ but not CD4^+^ T cells were virtually absent. *In vitro* analysis demonstrated a tumor-mediated elimination of CD8^+^ T cells that was contact dependent and involved the caspase-3 pathway. Most importantly, we found that the BCL1 cells expressed characteristics of B10 regulatory B cells, *i*.*e*., they were CD1d^hi^CD5^+^ and secreted high levels of IL-10. These BCL1 tumor cells can inhibit anti-tumor immune responses by depleting CD8^+^ effector T cells.

## Introduction

The tumor site represents a complex microenvironment where malignant cells reconstruct the milieu to promote tumor growth while anti-tumor immune responses aim to eliminate tumor cells. When effective, anti-tumor immunity can induce quiescence or dormancy in tumor cells. Cancer dormancy is a clinical state in which tumor cells are present in clinically healthy subjects but are maintained in a steady state; yet relapses can occur years later [1]. Indeed, many cancer patients who are in complete remission have circulating tumor cells (CTCs) in their blood or bone marrow [2, 3] and the presence of these cells suggests that tumor is present but probably dormant [4].

We have extensively studied tumor immunity in the mouse BCL1 model of tumor dormancy [5–7]. This is a relatively unique model where tumor dormancy is mediated by antibody against the idiotype (Id) of IgM/IgD expressed on the BCL1 cells. When inoculated into non-immunized BALB/c mice, the BCL1 tumor cells initially proliferate in the spleen, resulting in pronounced splenomegaly. Tumor cells are later found in blood and lymph nodes. Immunization with the BCL1 IgM-Id induces high titers of anti-Id antibodies that provide long-lasting protection against subsequent challenge with BCL1 tumor cells. Although the tumor cells grow for a short time in the immunized mice, tumors then regress and persist in a constant number (10^6^−10^7^) in the spleen. Over time, tumor cells can proliferate in some of the mice and this “relapse” has been attributed to several factors including decreases in anti-Id antibody titers and mutations in the signaling pathway by which the anti-Id inhibits tumor growth [7].

Cellular immunity against the BCL1 tumor can enhance dormancy as suggested by the fact that interferon-gamma (IFN-γ) secreted by Th1 cells or cells of the innate immune system prolong immunity mediated by anti-Id antibody [8]. However, effective immunity can also be induced in SCID mice treated with anti-Id antibodies, thus demonstrating that T cell-mediated cellular immunity is not absolutely required [9].

A formidable obstacle confronted in cancer therapy is the ability of tumor cells to evade anti-tumor immunity. This occurs partially through the tumor-mediated induction of suppressor cells within the tumor microenvironment. Immune-suppressor lymphocyte subsets such as regulatory T cells (T_regs_) and B cells (B_regs_) normally maintain systemic homeostasis by preventing autoimmunity and alleviating inflammation following the resolution of infection or injury [10–12]. However, tumor cells can manipulate these regulatory lymphocyte subsets to inhibit anti-tumor immune responses. Infiltration of T_regs_ in the tumor microenvironment has been reported to induce immune tolerance and thus promote tumor progression [13–16]. Moreover, increased numbers of T_regs_ have also been shown to correlate with a poor prognosis in cancer patients [16,17].

The mechanisms by which T_regs_ suppress effector immune responses include direct cell contact [18,19] and secretion of immunosuppressive cytokines [20–22]. B cells have also been shown to control immune hyperactivity primarily through the production of IL-10 [23]. Such immunomodulatory “B_regs_” can induce T_regs_, downregulate T_H_1 cytokines, and limit T_H_17 differentiation [24–27]. Therefore, B_regs_ may also contribute to tumor progression.

In this study, we examined the role of regulatory cells in tumor progression. In the BCL1 tumor model dormant *vs*. active tumor environments could be examined in parallel. Our aim was to determine whether the presence of anti-tumor immune responses diminished the putative role of T_regs_ on tumor progression. Unexpectedly, we found higher numbers of T_regs_ in the spleens of mice harboring dormant tumor cells, as compared to the spleens of their non-dormant counterparts where tumor cells were rapidly expanding. Yet, T_regs_ isolated from both tumor microenvironments suppressed the proliferation of effector T cells *in vitro* equally well. Our results show that in the BCL1 tumor, accumulation of T_regs_ in the tumor site did not directly correspond with tumor growth and thus may be only one correlate of disease progression. Furthermore, we observed that the BCL1 tumor cells exhibited the phenotype and cytokine profile of the B10 subset of B_regs_ and they directly suppressed CD8^+^ T cells. Therefore, the tumor cells were the most abundant inhibitory cell subset in the tumor microenvironment. Our results suggest that cross-talk between malignant B_regs_ and different types of normal effector T cells might be extremely important in the growth *vs*. stasis of B_reg_-like tumors, such as chronic lymphocytic leukemia (CLL), in humans. Although this is a single tumor model, it raises important questions about the interactions between B lymphoid tumors and both effector and regulatory T cells in their environment.

## Materials and Methods

### Mice

BALB/c mice were bred and maintained in the animal colony at the University of Texas Southwestern Medical Center (UTSW) at Dallas. This study was designed in strict accordance with the recommendations in the Guide for the Care and Use of Laboratory Animals of the National Institutes of Health and the protocol was approved by the UTSW Institutional Animal Care and Use Committee (IACUC). Animals were monitored daily by the animal facility caretakers and weekly by a staff veterinarian. Mice that became unexpectedly ill or exhibited signs of distress were removed from the study, anesthetized then sacrificed by cervical dislocation.

### Purification of the Id^+^ BCL1 IgM

A BCL1 hybridoma cell line (BCL1X x63) that secretes the Id^+^ IgM and IgG_1_ (MOPC-21) was used for generating and purifying the BCL1 IgM, as described previously (5). Cells were expanded in DMEM (Sigma; St. Louis, MI) supplemented with 10% fetal bovine serum (FBS) (Hyclone; Logan, UT) in a standard incubator (37°C, 5% CO_2_) for 5–7 days. The supernatants were collected and mixed with a saturated ammonium sulfate solution (Sigma) to a final concentration of 30% and total protein was allowed to precipitate overnight at 4°C. The precipitate was dissolved in Millipore water, and dialyzed [MWCO 12–14,000] (Spectrum Labs; Irving, TX) against PBS, pH 7.4. The IgM was purified by chromatography on an Ultrogel AcA 34 polyacrylamide matrix column (Sigma).

### Conjugation of the BCL1-IgM to Keyhole Limpet Hemocyanin (KLH)

BCL1 IgM and KLH (CalBiochem; Los Angeles, CA) were each adjusted to 1 mg/mL with PBS, combined at a 1:1 ratio, mixed with 1.25% glutaraldehyde (Fluka AG; New York, NY), and incubated for 4 hours with continuous mixing. The conjugate solution was dialyzed against PBS at pH 7.4 and aliquots were stored at -80°C.

### Emulsification of the BCL1/KLH conjugate in Complete Freund’s adjuvant (CFA) and immunization of mice

For each immunization, 100 μg BCL1-Id IgM/KLH was combined with an equal volume of CFA (100 μL) (Sigma), emulsified in a mixer (Spex; Metuchen, NJ), and drawn into a 1 cc glass tuberculin syringe (BD Biosciences) fitted with a 25 gauge x 5/8” needle (BD Biosciences; Franklin Lakes, NJ). Female BALB/c mice (10–14 weeks) were injected subcutaneously in their lower and upper flanks (100 μL/side) on D-45 and D-38, and under the nape of the neck (200 μL) on D-28 (relative to the day of tumor inoculation).

### BCL1 tumor inoculation

A frozen aliquot of spleen cells from BCL1 tumor-bearing mice was thawed and suspended in cold, sterile HBSS (Gibco; Gaithersburg, MD). Mice were injected with 5 x 10^4^ cells/mouse into the peritoneum 28 days (D0) after the third and final immunization.

### Examination of mice for dormancy or splenomegaly

Beginning at three weeks after the injection of tumor cells (D+21), mice were palpated twice weekly for splenomegaly. Mice were assigned a spleen index (SI) of 1–4 corresponding to increasing spleen size, as described previously [7]. Briefly, an SI = 1 corresponds to a normal spleen. A spleen that extends to the mid-line of the abdomen harbors dormant tumor cells and is assigned as SI < 2.5. Spleens that extend beyond the midline indicate active proliferation of the BCL1 tumor cells and have an SI > 2.5. Splenomegaly occurs between days 19 to 43 after injecting BCL1 tumor cells. No splenomegaly was detected in immunized mice at D+60 so this was established as a cutoff point for the designation of dormancy [28].

### Phenotypic analysis of leukocyte subsets by flow cytometry

Mice were sacrificed after 60 days and spleens, inguinal and mesenteric lymph nodes were harvested and homogenized into single cell suspensions using HBSS + 2% FBS (Gibco; Gaithersburg, MD). Spleen cells were further treated with ammonium chloride lysing solution to remove erythrocytes. The following anti-mouse antibodies were used for immunophenotyping: anti-CD1d (1D1) (BD Pharmingen; San Diego, CA), anti-CD3 (145-2C11) (BD Pharmingen), anti-CD4 (GK1.5) (eBioscience, San Diego, CA), anti-CD5 (53–7.3) (BD Pharmingen), anti-CD8 (53–6.7) (eBioscience), anti-CD11b (M1/70) (eBioscience), anti-CD19 (6D5) (Caltag Laboratories; Burlingame, CA), anti-CD25 (PC61) (BD Pharmingen), anti-Ter119 (TER-119) (eBioscience), anti-B220 (RA3-6B5) (BD Pharmingen), anti-DX5 (DX5) (eBioscience), anti-Gr-1 (RB6-8C5) (eBioscience), and anti-FoxP3 (FJK-16s) (eBioscience). Staining for FoxP3 was performed according to the manufacturer’s protocol (eBioscience). All samples were acquired on a FACSCalibur flow cytometer (BD Biosciences; San Jose, CA) and analyzed by FlowJo software (Ashland, OR).

### Purification of CD4^+^, CD8^+^, and T_reg_ cells from the spleens of donor mice

To purify CD4^+^ T cells and T_reg_ cells from spleens, erythrocytes were lysed and these cells were magnetically enriched following the manufacturer’s protocol (StemCell Technologies; Vancouver, Canada). To sort T_reg_ cells, the CD4^+^ -enriched spleen cells were further stained for cell surface markers using anti-CD4 (GK1.5), anti-CD25 (PC61), and a “lineage cocktail” consisting of anti-CD11b (M1/70), anti-B220 (RA3-6B5), and anti-Ter119 (TER-119). Cell sorting was performed on a FACSAria (BD Biosciences). To purify CD8^+^ cells, spleen cells were stained with a cocktail of biotin-labeled antibodies against non-CD8 cell markers [CD4 (RM 4.40), CD11b, B220, CD19, DX5, I-A/IE] and magnetically purified using streptavidin microbeads according to the manufacturer’s protocol (Miltenyi Biotec; Auburn, CA).

### T_reg_ cell suppression assays

Sorted T_regs_ were co-cultured in graded doses with purified “responder” CD4^+^, CD8^+^, or BCL1.3B3 cells, the *in vitro*-adapted cell line of BCL1 tumor cells [29]. Responder cells were cultured in 96-well plates (5 x 10^4^ cells/well) with irradiated BALB/c spleen cells as a source of antigen-presenting cells (50 Gy) and 4 μg/mL anti-CD3 (BioLegend; San Diego; CA) in RPMI medium (Gibco). For thymidine incorporation, 3-day cultures were pulsed overnight with 1 μCi [^3^H] thymidine (PerkinElmer; San Jose, CA), harvested, and counted in a scintillation counter (PerkinElmer). For CFSE dilution, responder cells were labeled with 5μM CFSE (Invitrogen) and co-cultured with T_regs_. When necessary, blocking antibodies against IL-10 [JES5-2A5] (BioLegend) and TGF-β [1D11] (R&D Systems; Minneapolis, MN) were added. For Transwell assays, T_reg_ cells were cultured on 0.4 μm inserts (BD Falcon; Bedford, MA).

### BCL1-mediated T cell suppression assays

Purified CD8^+^ cells were labeled with 5 μM CFSE (Molecular Probes; Eugene, OR) and co-cultured with irradiated BALB/c spleen cells (50 Gy), anti-CD3 (2 μg/mL; BioLegend), rIL-2 (50 ng/mL; Roche; Indianapolis, IN) and cultured in graded doses with BCL1 tumor cells in complete medium. BCL1 tumor cells were purified from inguinal lymph nodes of mice with a high tumor burden (SI > 2.5) using biotin-labeled rat anti-BCL1-Id IgM (clone 6A5, a gift from Dr. Freda Stevenson) and streptavidin-conjugated beads (Miltenyi Biotec). For Transwell assays, BCL1 tumor cells were cultured on 0.4 μm inserts (BD Falcon).

### Intracellular cytokine staining for IL-10

Spleen cells from mice bearing active BCL1 tumor cells and naïve mice were suspended at 1 x 10^6^ /mL in stimulation medium prepared using RPMI-1640 (Sigma), L-glutamine (Sigma), NEAA (Gibco), Na-pyruvate (Gibco), 10% heat-inactivated FBS β-mercaptoethanol (50 μM; Sigma), LPS (10 μg/mL; Sigma), PMA (Sigma), Ionomycin (500 ng/mL; Sigma), and monensin (2 μM; BioLegend) and incubated for 5 hours at 37°C, 5% CO_2_. Samples were washed with cold PBS+10%FBS and stained with antibodies against BCL1-Id, CD3 (145-2C11), CD11b (M1/70), Ter119 (TER119) [for tumor cells] and anti-CD19 (6D5) [normal B cells]. Cells were fixed and permeabilized (Perm Buffer; eBioscience) then stained with anti-IL-10 antibody [JES5-2A5] or isotype control (IgG2b; BioLegend) and analyzed on a FACSAria flow cytometer.

### Analysis of soluble cytokines and BCL1-Id by ELISA

Serial dilutions of supernatants from 3-day cultures of BCL1.3B3 tumor cells were added to ELISA plates (BD Falcon) coated with rat anti-BCL1-Id. A positive control (previously purified BCL1-Id from our laboratory stocks) was serially diluted and used to generate the standard curve. An Id-IgM myeloma protein of irrelevant specificity (MOPC-104E, Sigma) was used as the negative control. Rabbit anti-mouse IgM μ-chain-HRP (Jackson Immunoresearch; West Grove, PA) was used as the detection antibody with O-phenylenediamine dihydrochloride (OPD) substrate (Thermo Scientific Pierce; Waltham, MA) as the detection solution and plates were read on an ELISA plate reader (Molecular Devices; Sunnyvale, CA). The cytokines IL-1β, IL-4, IL-6, IL-10, TNF-α, and IFN-γ (R&D Systems), and TGF-β (eBioscience) were also quantified by sandwich ELISA according to the manufacturer’s protocol.

### Caspase-3 Activation

CD8^+^ T cells were purified from the spleens of BALB/c mice using magnetic beads according to the manufacturer’s directions (StemCell Technologies) and cultured with irradiated splenocytes, anti-CD3 (2 μg/mL; BioLegend), rIL-2 (50 ng/mL), and graded doses of purified BCL1 tumor cells. Anti-FasL (CD178, BioLegend) was added at 10 μg/mL as indicated. Cells were stained with fluorochrome-conjugated anti-CD8 (eBioscience) and anti-B220 (BD Biosciences) antibodies. Cells were then stained for intracellular caspase-3 (BD Pharmingen) according to the manufacturer’s specifications. Samples were acquired on a FACSCalibur flow cytometer and analyzed using FlowJo software.

## Results

### Immunization of BALB/c mice with the BCL1 Id^+^ IgM elicits the production of anti-Id antibody and results in the inhibition of tumor cell growth and the depletion of CD8^+^ T cells

**Fig 1:**
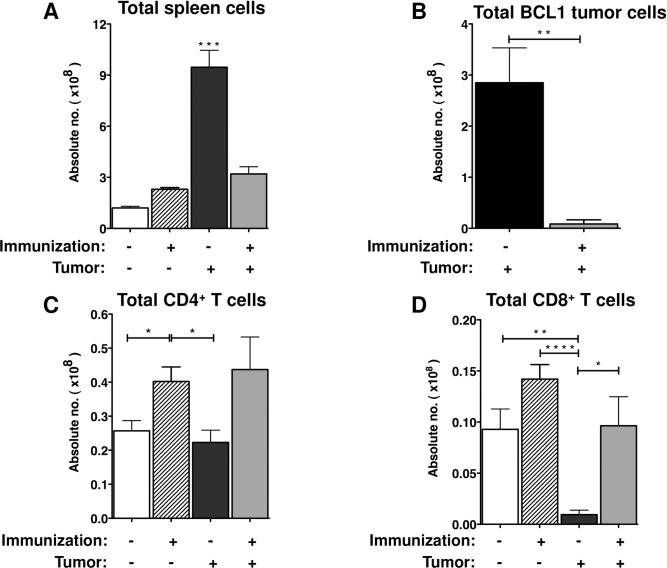
Increased BCL1 tumor cell burdens leads to the depletion of CD8^+^ T cells. Groups of mice immunized with the BCL1-Id along with non-immunized groups were inoculated with BCL1 tumor cells. Sixty days after tumor challenge, immunophenotyping was performed on spleen cells. (A) The total number of spleen cells from mice that were challenged with BCL1 tumor. (B) The total number of BCL1 tumor cells in the spleen. The total number of (C) CD4^+^ T cells, and (D) CD8^+^ T cells in the spleen from all experiment groups. Each group represents a mean of four to eight mice from at least 3 experiments. Data are shown as mean ± SEM (* *p* < 0.05, **p < 0.005, *** *p* < 0.0005, ****p < 0.0001; student’s t-test).

Immunization of mice with the BCL1-Id^+^ IgM-KLH conjugate resulted in the production of high titers of anti-Id antibodies, as has been shown previously [30]. Serum titers of anti-Id measured on D+60, the time-point for dormancy, from mice bearing dormant BCL1 tumors were 13-fold higher than those with non-dormant tumor cells on (data not shown). Mice bearing non-dormant tumor cells had significantly more spleen cells than their dormant counterparts (9.5 x 10^8^ *vs*. 3.4 x 10^8^ respectively, *p* = 0.0002) (Fig 1A). The BCL1 tumor cells accounted for the difference in the numbers of spleen as mice with non-dormant tumors cells had significantly higher numbers of BCL1 tumor cells than those harboring dormant tumor cells (2.9 x 10^8^ *vs*. 8.5 x 10^6^, respectively, *p* = 0.001) (Fig 1B).

We also examined levels of CD4^+^ and CD8^+^ T cells in the spleens on D+60. Immunization alone resulted in a significant increase in the total number of CD4^+^ T cells (4.02 x 10^7^ cells, *p* = 0.032) relative to controls (2.57 x 10^7^ cells) (Fig 1C) and a modest but not statistically significant increase in the total number of CD8^+^ T cells (1.42 x 10^7^ cells *vs*. 0.93x10^7^ cells, *p* = 0.092) (Fig 1D). In the absence of immunization, the robust proliferation of BCL1 tumor cells in the spleen correlated with in an almost complete elimination of CD8^+^ T cells relative to controls (9.9-fold reduction, *p* = 0.001) (Fig 1D). However, CD4^+^ T cells did not experience a statistically significant reduction (1.1-fold change, *p* = 0.545) (Fig 1C). In contrast, both the CD4^+^ and CD8^+^ T cells in the spleens of mice with dormant tumor remained stable (Fig 1C and 1D). Therefore, active proliferation of tumor cells leads to the elimination of CD8^+^ T cells from the tumor site. In contrast, dormant tumor cells do not cause a depletion of CD8^+^ T cells from the tumor site.

### Quantification of T_reg_ cells in the spleens of mice with dormant tumor

**Fig 2:**
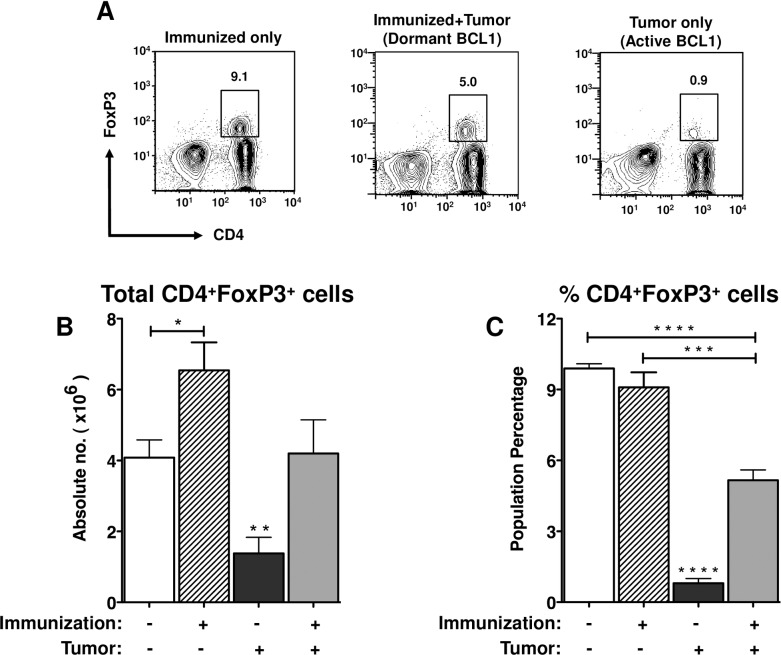
Immunization results in an increase in T_regs_ while BCL1 tumor cell growth reduces T_regs_ at the tumor site. (A) Spleen cells from mice immunized with BCL1-Id and inoculated with BCL1 tumor cells were examined for T_regs_ (CD4^+^Foxp3^+^) 60 days after tumor challenge. (B) total number, and (C) percentage of T_regs_ in the spleen were determined based on the total viable spleen cells. The values are a compilation of at least 3 different experiments with 4–7 mice per group. Representative plots include 8 or more mice per group. Data are shown as mean ± SEM (* *p* < 0.05, **p < 0.005, *** *p* < 0.0005, ****p < 0.0001; student’s t-test).

It has been reported that T_regs_ infiltrate tumor sites in a wide variety of cancers [13–16]. On D+60 we examined the numbers of T_regs_ in the spleens of mice with dormant *vs*. actively growing BCL1 tumor cells and the corresponding controls and found notable differences in numbers of T_regs_. (Fig 2A). We observed the following: 1. Immunization alone resulted in a significant increase in the total number of T_regs_ (Fig 2B) but their percentages remained similar to those observed in the spleens from control mice (Fig 2C). 2. The subsequent challenge of immunized mice with BCL1 tumor cells resulted in a reduction in the number of T_regs_ in the spleen, *i*.*e*. spleens from mice with both dormant and actively growing tumor had fewer T_regs_ (4.2 x 10^6^ cells, *p* < 0.07 and 3.2 x 10^6^ cells, *p* = 0.0002, respectively) than mice that were immunized but not injected with tumor cells (6.5x10^6^ cells) (Fig 2B). 3. All mice (with or without immunization) that were inoculated with tumor cells experienced a reduction in T_regs_ in their spleens relative to their respective controls. T_regs_ were fewest in mice that received BCL1 tumor cells without prior immunization (1.4 x 10^6^ cells). In this group, T_regs_ in the spleen constituted only 0.8% of the total lymphocytes compared to 9.9% in control mice (Fig 2C). 4. Overall, the number of T_regs_ decreased as tumor cells rapidly proliferated in the spleens, suggesting that rapid tumor cell expansion leads to the depletion of T_regs_ at the tumor site.

### The presence of BCL1 tumor cells in inguinal lymph nodes has no effect on the numbers of different T cells

**Fig 3:**
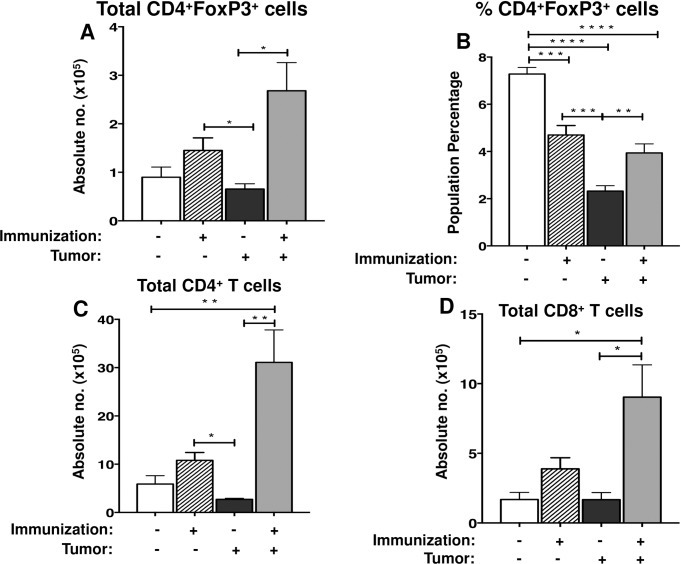
BCL1 tumor cells do not impact regulatory and conventional T cells in the draining lymph nodes. (A) The total number and (B) percentage of T_regs_ (CD4^+^Foxp3^+^) in the inguinal lymph nodes were examined within each group of mice 60 days after tumor challenge. Healthy control mice were age-matched to the experiment groups. The total number of (C) CD4^+^ T cells and (D) CD8^+^ T cells within the inguinal lymph nodes were determined within each group at 60 days after tumor challenge. The values represent a compilation of 1–3 different experiments with 4–13 mice per group. Data are shown as mean ± SEM (* *p* < 0.05, **p < 0.005, *** *p* < 0.0005, ****p < 0.0001; student’s t-test).

It has been well documented that spleens contain far more BCL1 cells than other organs even when tumor dormancy is established [28]. Nevertheless, we examined T cells subsets in the draining lymph nodes to determine if they were also altered by the presence of tumor cells. We found that immunization alone resulted in an increase in T_regs_ in the inguinal lymph nodes. The percentage of the T_regs_ in the inguinal nodes of all mice correlated with their spleens. (Fig 3A and 3B). However, unlike the situation in the spleen, the presence of small numbers of BCL1 tumors in the inguinal lymph nodes did not lead to a significant reduction of T_regs_ as compared to healthy controls. Both the CD4^+^ and CD8^+^ T cells in the inguinal lymph nodes were also boosted by immunization. (Fig 3C and 3D). This was likely due to the fact that these nodes drained the sites of injection.

We also enumerated the T cell subsets in the mesenteric lymph nodes and found no difference in any of the experimental groups examined (data not shown). These results support the hypothesis that a certain threshold of tumor cells is required before T cells are depleted.

### Immunization with BCL1-Id does not impair the function of T_regs_ in the tumor microenvironment

**Fig 4:**
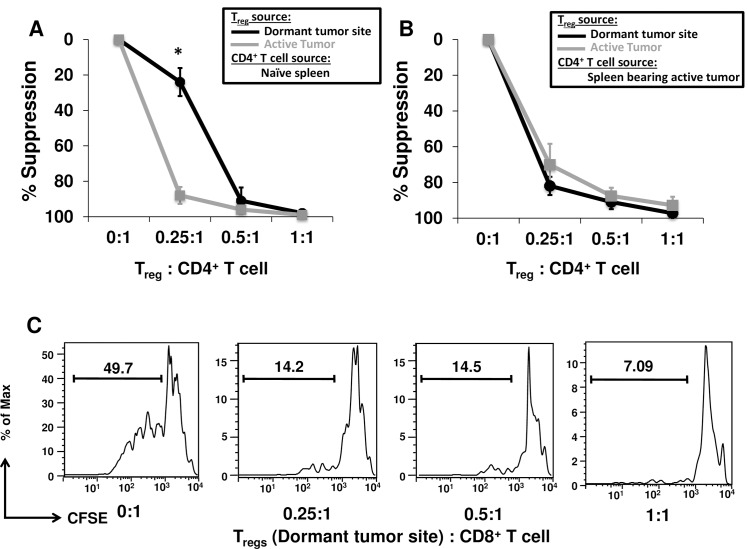
T_regs_ from the dormant BCL1 tumor microenvironment maintain their capacity to suppress CD4^+^ and CD8^+^ T cells. T_reg_ suppression assays were performed using CD4^+^ T cells purified from spleens of (A) naive mice, or (B) BCL1 tumor-inoculated mice. Suppression by T_regs_ was calculated relative to the samples containing CD4^+^T cells only (0:1). The data are shown as mean ± SEM from three experiments (* *p* < 0.05). (C) T_regs_ were sorted from spleens of mice harboring dormant BCL1 tumor cells and co-cultured with CFSE-labeled CD8^+^T cells purified from naive mice. Histogram plots are gated on CD8^+^ cells and the values represent the percent CFSE dilution. Representative plots of 3 independent experiments yielding similar results.

Since T_regs_ were more prevalent in the spleens from mice with dormant tumor cells as compared to those with actively growing tumor cells, we examined their capacity to inhibit effector T cell proliferation using T_reg_ suppression assays. We observed that T_regs_ from mice with either dormant or actively growing tumor suppressed the proliferation of naïve CD4^+^ T cells (Fig 4A). However, T_regs_ from spleens of mice with actively growing tumor were more potent suppressor cells than those from spleens with dormant tumor cells *i*.*e*. they inhibited the proliferation of CD4^+^ T cells at the lowest ratio (0.25:1). Since the tumor microenvironment can impair effector T cell function, we also isolated CD4^+^ T cells from the spleens of mice with actively growing tumor mice to use as responder cells. In this case, T_regs_ from both BCL1 tumor environments performed similarly in their ability to suppress the proliferation of CD4^+^ T cells suggesting that the tumor microenvironment also contributes to the functionality of effector T cells (Fig 4B).

T_regs_ from mice with dormant tumor cells also inhibited the proliferation of CD8^+^ T cells, further demonstrating that the induction of dormancy did not impair the function of T_regs_. Importantly, T_regs_ almost completely prevented the proliferation of CD8^+^ T cells in co-culture (Fig 4C). T_reg_-induced suppression of CD8^+^ T cells was also observed when their numbers were adjusted to match the physiological ratios we observed in the spleen (average of 0.4-to-1 from Figs 1D and 2B). Together, these data suggest that T_regs_ from the mice with both dormant and actively growing tumors suppress the proliferation of CD4^+^ and CD8^+^ T cells to the same extent, but that there were more T_regs_ in the spleens of mice with dormant tumors.

### T cell suppression by T_regs_ requires cell contact

**Fig 5:**
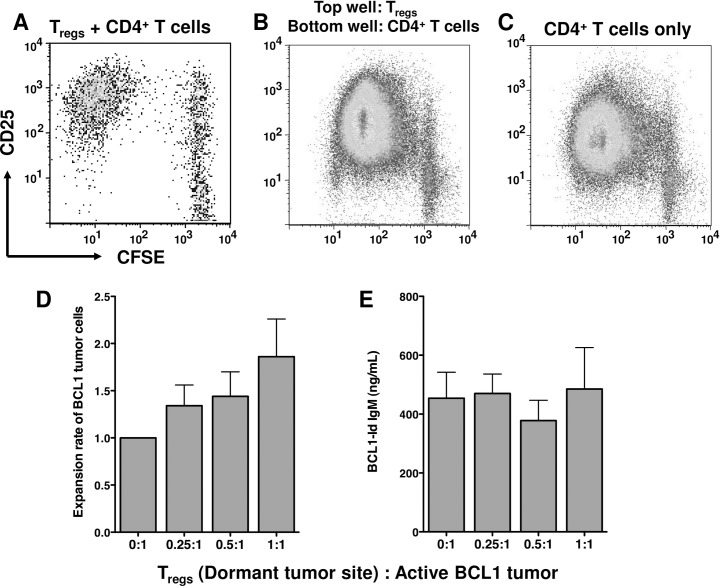
T_regs_ suppress CD4^+^ T cell proliferation by cell contact but do not suppress BCL1 tumor cells. (A) T_regs_ from mice harboring dormant BCL1 tumor cells were cultured in equal numbers with CFSE-labeled CD4^+^ T cells. (B) T_regs_ were plated onto trans-well inserts and placed in wells containing CFSE-labeled CD4^+^ T cells in an equal ratio. (C) CFSE-labeled CD4^+^ T cells were assessed for proliferation in the absence of T_regs_. Plots are representative of 3 or more experiments yielding similar results. (D) Graded numbers of T_regs_ from mice with dormant BCL1 tumor cells were co-cultured with BCL1.3B3 tumor cells. Proliferation of BCL1.3B3 cells in the absence of T_regs_ (0:1) was considered to be the baseline values. Data represent the average of 3 experiments. *E*, BCL1-Id^+^ IgM secreted by BCL1.3B3 cells as measured by ELISA. Data represent the average of 4 experiments.

We examined the mechanism by which T_regs_ suppress CD4^+^ T cells. Previous reports have shown that different subsets of T_regs_ can mediate suppression by the secretion of inhibitory cytokines such as IL-10, TGF-β and IL-35 and by direct cell contact [18]. In agreement with other reports [22, 31], our experiments showed that neither the addition of neutralizing anti-IL-10 antibodies nor rIL-10 decreased the *in vitro* suppressive activity of T_regs_ (data not shown). Moreover, addition of recombinant IL-10 did not suppress the proliferation of the CD4^+^ cells *in vitro* (data not shown). As noted earlier, co-culture of T_regs_ with CFSE-labeled CD4^+^ T cells inhibited their proliferation as observed by the lack of CFSE dilution (Fig 5A). However, in trans-well assays in which contact between T_regs_ and CD4^+^ T cells was prevented, T_reg_-induced suppression was lost (Fig 5B) and the proliferation of CD4^+^ T cells proliferation was similar to that of CD4+ cells in wells containing no T_regs_ (Fig 5C). These results show that T_regs_ from the spleens of mice with dormant tumors are functional and maintained their ability to suppress T cells and that suppression requires cell contact.

### T_regs_ cells do not suppress BCL1 tumor cell proliferation or IgM secretion *in vitro*

Most studies on T_reg_ function have examined their capacity to suppress T cell proliferation. However, it has also been shown that T_regs_ can directly suppress the proliferation of B cells [24]. Since the immunized mice had a higher ratio of T_regs_ to BCL1 tumor cells as compared to non-immunized tumor bearing mice, we examined the ability of T_regs_ to inhibit the proliferation of tumor cells. The baseline proliferation of BCL1 tumor cells in culture (no T_regs_ added) was measured and used to determine the relative proliferation of BCL1 tumor cells that were co-cultured with T_regs_. We found that T_regs_ co-cultured with equal numbers of BCL1 tumor cells, neither inhibited tumor cell proliferation (Fig 5D) nor induced a statistically significant change in the number of tumor cells.

We next examined the effects of T_reg_ cells on the secretion of IgM by BCL1 tumor cells. T_regs_ from the spleens of mice with dormant tumors were co-cultured with tumor cells for 3 days after which the media were collected and assayed by ELISA for levels of BCL1-Id^+^ IgM. As shown in Fig 5E, the addition of T_regs_ did not inhibit the secretion of IgM by the tumor cells. Therefore, T_regs_ within the tumor site did not exhibit any direct effects on BCL1 tumor cells that we could measure *in vitro*.

### BCL1 tumor cells exhibit characteristics of B_regs_

**Fig 6:**
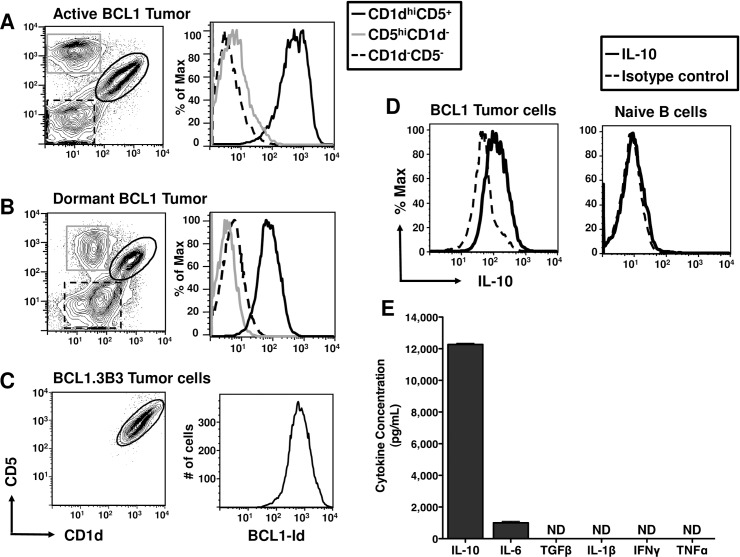
BCL1 tumor cells express the characteristics of B10 B_regs_. Examination of a B10 B_reg_ phenotype performed on spleen cells from mice injected with (A) BCL1 tumor cells only, (B) pre-immunized with BCL1-Id^+^ IgM and challenged with BCL1 tumor cells, and (C) *in* vitro-adapted BCL1 tumor cells. The analysis shows 3 distinct subsets, of which the B10 B_reg_ phenotype (CD1d^hi^CD5^+^) is unique to the BCL1-Id^+^ tumor cells. Representative plots of at least 3 or more independent experiments are shown. (D) Intracellular cytokine staining of spleen cells from mice challenged with BCL1 tumor cells (solid line) or naïve mice (dashed line). Histogram plots are representative of 3 independent experiments. (E) The cytokine profile of supernatants from BCL1.3B3 tumor cells following a 72 h culture period as determined by ELISA. Total values are shown as mean ± SEM from 2–3 experiments. ND, below detection limits.

B_regs_ are specialized B cells that secrete high levels of IL-10 and can suppress immune activation [32]. We hypothesized that since the BCL1 tumor cells are malignant B cells, and that they are immunosuppressive in mice [33], they might exhibit characteristics of B_regs_. To explore this possibility, we stained spleen cells from the BCL1 tumor-bearing mice with antibodies against BCL1-Id, and the cell surface markers, CD1d and CD5 that identify the B10 subset of B_regs_ [23]. An examination of the co-expression of CD1d and CD5 by flow cytometry yielded 3 distinct subsets of B cells in the spleen (Fig 6A). Of the 3 subsets, only the CD1d^hi^CD5^+^ subset, which corresponds to B10 B_regs_, also co-stained with the anti-BCL1-Id. Moreover, a similar B_reg_ phenotype was also observed using in the spleen cells from mice with dormant vs. actively dividing BCL1 tumor (Fig 6B), suggesting that immunization does not alter the B_reg_ phenotype of the tumor cells. Moreover, we observed that the BCL1.3B3 cell line (adapted for *in vitro* growth) [29] expressed the same B_reg_ phenotype (Fig 6C).

### Although BCL1 tumor cells secrete IL-10 they mediate T cell suppression primarily by inhibiting cell-cell contact

We next examined the tumor cells for secretion of IL-10, the signature CD1d^hi^CD5^+^ B_reg_ cytokine [32]. Intracellular staining of BCL1 cells directly harvested from spleens and showed that they indeed produced IL-10. (Fig 6D). By comparison, normal splenic B cells did not produce measurable levels of IL-10, confirming that IL-10 production is confined to the BCL1 tumor cells. We further studied the cytokine profile of the tumor cells by examining levels of an array of cytokines by ELISA in cultured BCL1.3B3 cells. We found that BCL1.3B3 cells secreted high levels of IL-10, but not TGF-β another immunosuppressive cytokine, or the pro-inflammatory cytokines, IFN-γ or TNF-α (Fig 6E). Interestingly, the BCL1 tumor cells also secreted IL-6, which has been described to contribute to the impairment of immune responses in the tumor milieu [34].

**Fig 7:**
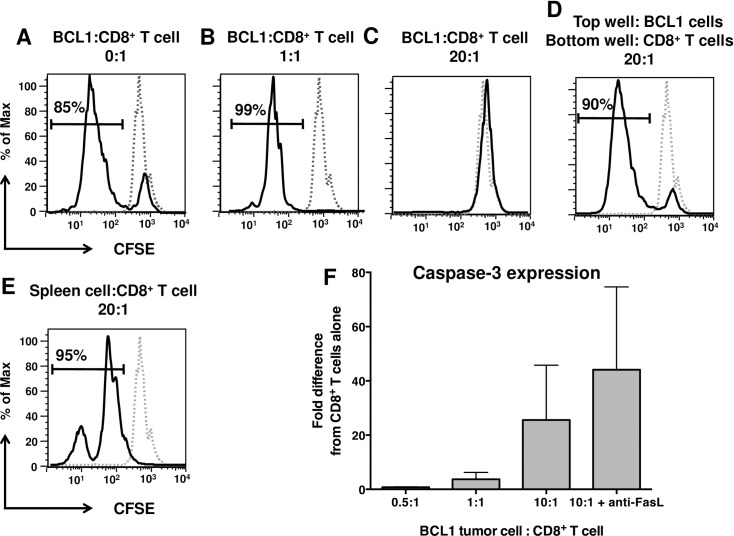
BCL1 tumor cells suppress the proliferation of CD8^+^ T cells in a contact-dependent manner. The proliferation of CFSE-labeled CD8^+^ T cells was determined (A) alone, or (B) with equal numbers of BCL1 tumor cells (1:1) or (C) High ratios of BCL1 tumor cells to CD8^+^ T cells, as observed in the tumor milieu. (D) BCL1 tumor cells were prevented from making contact with CD8^+^ T cells by using Trans-well inserts. *E*, Addition of large numbers of spleen cells from naïve mice did not inhibit the proliferation of CD8^+^ T cells. *F*, Assessment of caspase-3 levels in CD8^+^ T cells following co-culture with graded doses of BCL1 tumor cells. Values in FACS plots represent the percentage of CD8^+^ T cells undergoing proliferation. Representative plots of 3 independent experiments are shown.

As discussed earlier, CD8^+^ T cells were completely eliminated from the spleens of non-immunized BCL1 tumor-bearing mice (Fig 1D). Since the tumor cells expressed the phenotype and cytokine profile of B10 B_regs_, we hypothesized that they either inhibited the proliferation of CD8^+^ T cells or actively killed them. To further explore this issue, we modified the standard T_reg_ suppression assay by using the BCL1 tumor cells as suppressor cells. Purified BCL1 Id^+^ tumor cells were co-cultured in graded doses with CFSE-labeled CD8^+^ T cells for 72 hours. Cultures were analyzed for the proliferation of CD8+ cells using CFSE dilution. In the absence of BCL1 tumor cells, the majority of the CD8^+^ T cells underwent proliferation *in vitro* (Fig 7A). Interestingly, co-culturing CD8 T cells with equal numbers of tumor cells increased the proliferation of CD8^+^ T cells (Fig 7B). However, when examining the *in vivo* tumor microenvironment, we had observed that the number of BCL1 tumor cells was consistently >200-fold higher than the number of CD8^+^ T cells (Fig 1B and 1D). Therefore, for the *in vitro* assay, we adjusted the number of tumor and CD8^+^ T cells to numbers that would be more analogous to their physiological ratios. This resulted in a complete inhibition of the proliferation of the CD8^+^ T cells (Fig 7C). Taken together, these results lead us to hypothesize that when there are few tumor cells present, that they actually enhance the numbers of anti-tumor effector T cells. As the number of tumor cells increase and greatly outnumber these effector T cells, they can then kill them or inhibit their proliferation.

We also determined whether the T cell suppression was due to cell contact using the trans-well assay. We found that preventing contact between tumor cells and CD8^+^ T cells restored the proliferation of the latter (Fig 7D). This was not due to overcrowding in the cell culture since equal numbers of normal spleen cells had no such effect, although there were fewer rounds of cell division, as might be expected (Fig 7E). These results demonstrate that BCL1 tumor cells suppress the proliferation of CD8^+^ T cells primarily by cell-cell contact.

### BCL1 tumor cells induce apoptosis in CD8^+^ T cells by activating caspase-3

To gain further insight into the mechanism by which BCL1 tumor cells eliminate CD8^+^ T cells, we examined caspase-3 activation in the CD8^+^ T cells following their co-culture with BCL1 tumor cells. Caspase-3 is a member of the family of effector caspases and its active form triggers the apoptosis pathway. Caspase-3 activation can be initiated in target cells by the binding of Fas-ligand (FasL) to its surface receptor, CD95 (Fas), which leads to subsequent activation of the caspase-3-mediated apoptosis [35]. Since BCL1 tumor cells express FasL (data not shown), we questioned whether tumor cell-mediated inhibition of CD8^+^ T cells was induced *via* the FasL-mediated caspase cascade. We therefore co-cultured purified CD8^+^ T cells with graded doses of BCL1.3B3 tumor cells for 5 or 48 time hours and assessed the activation of caspase-3 in CD8^+^ T cells by intracellular staining and flow cytometry. Our results show that there was no difference in caspase-3 activation at either 5 or 48 hours (data not shown). However, casapase-3 activation increased in CD8^+^ T cells when they were cultured with larger numbers of BCL1.3B3 tumor cells (10:1) demonstrating a clear trend of increased caspase-3 activation in CD8+ T cells cultured with larger numbers of BCL1 tumor cells (Fig 7F). Although adding anti-FasL antibody to the cultures did not inhibit or reduce levels of activated caspase-3. This might be due to limitations of *in vitro* tissue culture assays in blocking molecular signaling pathways. However, we cannot rule out a role for other death domain ligands such as TRAIL, which also trigger the caspase cascade. Regardless, our results suggest that BCL1 tumor cells inhibit CD8^+^ T cells at least in part by caspase-3-mediated apoptosis.

## Discussion

In this study we used an established mouse model of B cell lymphoma, BCL1, to examine the interaction between tumor cells and T lymphocyte subsets within the tumor microenvironment, *i*.*e*. the spleen. Inoculation of tumor cells into BALB/c mice results in rapid growth of the tumor in the spleen over a period of 2–4 months. The tumor cells finally spill out into the blood and lymph nodes [6,28]. Immunization of mice using the IgM-Id antibody elicits a potent and specific antibody-mediated anti-tumor response. When immunized mice are inoculated with BCL1 tumor cells, the tumor grows for a short period of time and then regresses into a dormant state that can last for the lifetime of the mice [36]. CD8^+^ T cells and IFN-γ can prolong tumor dormancy [10]. However, little is known about the role of subsets of T cells in the dynamic growth and/or dormancy of the tumor. Furthermore, it is unclear whether malignant BCL1 cells themselves have any effect on these T cells.

In order to gain insight into the role of T cells in the BCL1 tumor model, we studied T cell subsets *in vivo* and their activity as both effector cells and target cells. The major findings to emerge from this study are: 1. Immunization against the BCL1-specific antigen preserved the levels of CD4^+^, CD8^+^, and T_reg_ T cell subsets even after tumor challenge. 2. Non-immunized mice with large tumor burdens had significantly fewer CD8^+^ T cells and T_regs_ in their spleens while the numbers of CD4^+^ T cells were normal. 3. Although T_regs_ were present in the spleens of tumor-bearing immunized mice, tumor growth never progressed to clinical disease. 4. There were small numbers of tumor cells in lymph nodes but the T cell numbers remained normal in all the different groups of mice. 5. As determined by *ex vivo* experiments, T_regs_ from the spleens of mice with dormant tumor cells had normal suppressive activity but did not inhibit the proliferation of, or IgM secretion by the tumor cells suggesting that they do not act directly on the tumor cells themselves. 6. The BCL1 tumor cells had the phenotype and cytokine profile of normal B10 B_regs_. 7. BCL1 tumor cells suppressed the proliferation of CD8^+^ T cells *in vitro* and this suppression was dependent upon cell-cell contact and the likely involved activation of caspase-3.

Taken together, these results suggest that CD8^+^T cells in the spleen are stimulated to expand by small numbers of tumor cells and initially keep them in check. As the tumor cells outgrow the cytotoxic T cells, they eventually kill them by cell-cell contact. In contrast, when anti-Id is present the tumor cells are prevented from expanding at a rapid rate. As a result, the cytotoxic T cells kill them and the tumor cells therefore proliferate and are killed at the same rate leading to a steady state that we define as dormancy. If/when the titers of anti-Id wane, the tumor cells grow rapidly and escape dormancy by once again killing effector T cells and T_regs_. Thus, there is a complex and time-dependent interplay between the neoplastic B_regs_ (which are cytotoxic for the effector T cells and T_regs_), the anti-Id antibody and CD8^+^ cells (which are cytotoxic for the tumor cells), and the T_regs_, (which kill the tumor-reactive CD8^+^ T cells) in the induction and maintenance of dormancy.

The major difference between mice harboring dormant *vs*. actively growing BCL1 tumor cells is that CD8^+^ T cells are virtually eliminated and T_regs_ are reduced in number in the latter situation. *In vitro* studies also demonstrated that large numbers of BCL1 cells suppress the growth of CD8^+^ T cells in a contact-dependent manner. Since BCL1 tumor cells express Fas-Ligand, binding to Fas on target cells likely initiates caspase-mediated apoptosis [35]. Our co-culture assays showed that high ratios of BCL1 tumor cells were needed to induce caspase-3 activation in CD8^+^ T cells suggesting that prolonged or repeated contact between BCL1 tumor cells and cytotoxic T cells might be required to kill the latter. This is consistent with our hypothesis that large numbers of BCL1 tumor cells are needed to eliminate cytotoxic T cells. T_regs_ and CD8^+^ T cells were not depleted from the inguinal lymph nodes, likely due to their small numbers and suggesting that the tumor-induced immunosuppressive environment was confined to the spleen. Hence, in actively growing tumors, effector T cells would be eliminated. In contrast, in dormant tumor or in tumor localized in lymph nodes, the tumor cells would not be present in sufficient numbers to kill all the effector cells. Thus, dormancy actually induces a steady state where the BCL cells divide and are killed at the same rate due to an "optimal" ratio of tumor cells to T cells. Although soluble IL-10 secreted by the BCL1 tumor cells alone was insufficient to prevent T cell proliferation it likely contributed to an immunosuppressive milieu.

Our results are consistent with those from Saito *et al*. who examined cells from patients with advanced melanoma [37]. In that report a significant loss of CD8^+^ T cells relative to other lymphocyte subsets was observed. They further noted that co-incubation of T cells with tumor cells led to a variety of functional impairments resulting in the activation of caspases and T cell apoptosis [37].

Taken together with these results, our studies demonstrate that rapidly proliferating tumor cells can kill cytotoxic and regulatory T cells in the tumor milieu. In contrast, BCL1 tumor cells, whether dormant or active, had no inhibitory effects on CD4^+^ T cells in the spleen. Total CD4^+^ T cells in the spleens of mice with active tumor growth were not significantly different than those in mice with dormant BCL1 tumor or no tumor. These results are consistent with those of Farrar *et al*. who showed that cellular anti-BCL1 responses did not require CD4^+^ T cells [8]. They also demonstrated that CD8^+^ but not CD4^+^ T cells contributed to the maintenance of BCL1 tumor dormancy. This might be explained by the possibility that subsets of CD4^+^ cells can express different levels of Fas and thus have varying responses to Fas [38]. Tuft *et al*. also reported that activation of cytotoxic T cells was independent of CD4^+^ T cells in several models of B lymphoma, including BCL1 [39]. Moreover, both studies showed that depletion of CD4^+^ T cells did not abrogate anti-BCL1 immunity. Hence, while CD4^+^ T cells are likely important for the initial activation of CD8^+^ T cells, once the latter are activated the former are no longer needed. That in turn would infer that APCs for memory CD8^+^ effector T cells are the tumor cells themselves or other APCs such as macrophages. This is consistent with a report describing the activation of memory-effector cytotoxic T cells [39].

An important finding to emerge from our study is that mice with large BCL1 tumor burdens also had fewer T_regs_ in their spleens than mice harboring dormant BCL1 tumor cells. Our results differ from those using other tumor models where increases in T_regs_ correlate with tumor expansion and disease progression. Other studies have suggested that T_regs_ support tumor growth by inhibiting anti-tumor immunity [40]. Moreover, clinical studies have reported increased numbers of T_regs_ in tumors and draining lymph nodes in patients with solid tumors [13,16]. Yet, while the presence of greater numbers of T_regs_ has been associated with the progression of solid tumors, increased numbers of T_regs_ in patients with hematologic tumors does not always confer poor clinical outcomes [41,42]. Therefore, it is possible that rapidly growing neoplastic B_regs_ are unique in their ability to suppress or kill T_regs_. However, the T_reg_ profile that we describe here may also be unique to this particular model of B cell lymphoma and tumor dormancy. Although we found that the T_regs_ had no effects on BCL1 tumor cells *in vitro* even though they have been reported to directly suppress the activation of normal B cells [43], the activation of normal B cells versus proliferation of malignant B cells represent two very different situations.

The most intriguing finding was that the BCL1 tumor cells expressed the phenotype (CD1d^hi^CD5^+^) and cytokine profile (IL-10^+^) of B10 B_regs_. These cells have been reported in both mice and humans [44,45]. Human B_regs_ are also CD19^+^CD1d^hi^CD5^+^ and are functionally immunosuppressive. They have been reported to inhibit autoimmune disorders [46]. A recent study further elucidated B regulatory functions by demonstrating shared characteristics between CLL, a common adult B cell leukemia, and human IL-10^+^ B_regs_ [47]. Notably, the ability of CLL cells to produce IL-10 appeared to influence disease progression [23] probably, in part, by immunosuppression. Indeed patients with CLL often succumb to infections. Interestingly, the clinical characteristics and histopathology of BCL1 tumor cells were described to be analogous to prolymphocytic leukemia or CLL in humans [48,49].

The data presented in this study offer new insights into the presence and function of normal T_regs_ and malignant B_regs_. While the major mechanism for the establishment of dormancy appears to be signaling by anti-Id antibody [9], anti-Id does not eliminate all the tumor cells. However, once most of the tumor cells are eliminated, the interplay between the remaining effector and regulatory T cells and the tumor cells leads to the steady state that we define as "dormancy". When tumor “relapse” occurs, the tumor cells again grow, kill the effector T cells, immunosuppress the host (likely by IL-10 secretion), and result in death. Our results support the strategy of combining inhibitory anti-tumor antibodies or immunoconjugates with antibodies or drugs that can unleash the effector T cells in the host.
